# Systemic administration of mesenchymal stem cells combined with parathyroid hormone therapy synergistically regenerates multiple rib fractures

**DOI:** 10.1186/s13287-017-0502-9

**Published:** 2017-03-09

**Authors:** Doron Cohn Yakubovich, Dmitriy Sheyn, Maxim Bez, Yeshai Schary, Eran Yalon, Afeef Sirhan, May Amira, Alin Yaya, Sandra De Mel, Xiaoyu Da, Shiran Ben-David, Wafa Tawackoli, Eric J. Ley, Dan Gazit, Zulma Gazit, Gadi Pelled

**Affiliations:** 1 Skeletal Biotech Laboratory, Hebrew University-Hadassah Faculty of Dental Medicine, Jerusalem, 91120 Israel; 2 Department of Surgery, Cedars-Sinai Medical Center, Los Angeles, CA 90048 USA; 3 Board of Governors Regenerative Medicine Institute, Cedars-Sinai Medical Center, Los Angeles, CA 90048 USA; 4 Biomedical Imaging Research Institute, Cedars-Sinai Medical Center, Los Angeles, CA 90048 USA; 5 Division of Trauma and Critical Care, Cedars-Sinai Medical Center, Los Angeles, CA 90048 USA; 6 Department of Orthopedics, Cedars-Sinai Medical Center, Los Angeles, CA 90048 USA

**Keywords:** Systemic stem cell administration, Parathyroid hormone, Rib fracture

## Abstract

**Background:**

A devastating condition that leads to trauma-related morbidity, multiple rib fractures, remain a serious unmet clinical need. Systemic administration of mesenchymal stem cells (MSCs) has been shown to regenerate various tissues. We hypothesized that parathyroid hormone (PTH) therapy would enhance MSC homing and differentiation, ultimately leading to bone formation that would bridge rib fractures.

**Methods:**

The combination of human MSCs (hMSCs) and a clinically relevant PTH dose was studied using immunosuppressed rats. Segmental defects were created in animals’ fifth and sixth ribs. The rats were divided into four groups: a negative control group, in which animals received vehicle alone; the PTH-only group, in which animals received daily subcutaneous injections of 4 μg/kg teriparatide, a pharmaceutical derivative of PTH; the hMSC-only group, in which each animal received five injections of 2 × 10^6^ hMSCs; and the hMSC + PTH group, in which animals received both treatments. Longitudinal in vivo monitoring of bone formation was performed biweekly using micro-computed tomography (μCT), followed by histological analysis.

**Results:**

Fluorescently-dyed hMSCs were counted using confocal microscopy imaging of histological samples harvested 8 weeks after surgery. PTH significantly augmented the number of hMSCs that homed to the fracture site. Immunofluorescence of osteogenic markers, osteocalcin and bone sialoprotein, showed that PTH induced cell differentiation in both exogenously administered cells and resident cells. μCT scans revealed a significant increase in bone volume only in the hMSC + PTH group, beginning by the 4^th^ week after surgery. Eight weeks after surgery, 35% of ribs in the hMSC + PTH group had complete bone bridging, whereas there was complete bridging in only 6.25% of ribs (one rib) in the PTH-only group and in none of the ribs in the other groups. Based on the μCT scans, biomechanical analysis using the micro-finite element method demonstrated that the healed ribs were stiffer than intact ribs in torsion, compression, and bending simulations, as expected when examining bone callus composed of woven bone.

**Conclusions:**

Administration of both hMSCs and PTH worked synergistically in rib fracture healing, suggesting this approach may pave the way to treat multiple rib fractures as well as additional fractures in various anatomical sites.

**Electronic supplementary material:**

The online version of this article (doi:10.1186/s13287-017-0502-9) contains supplementary material, which is available to authorized users.

## Background

Affecting 350,000–400,000 patients each year in the United States, rib fractures occur in 10–15% of patients who sustain blunt trauma and are associated with morbidity and mortality rates in up to 25% of these patients [1]. Common complications are respiratory failure due to lung contusions and impaired mechanical properties as well as severe pain originating from the fractured rib. Patients rate their pain on average as 9.2 on a 1–10 scale and may not be able to return to work for at least 8.5 weeks [2]. Even after they return to daily activities, 67% of patients have reported prolonged disability and pain [3]. In patients suffering the most severe complications, poor mechanical ventilation leads to pneumonia, which is a frequent cause of death [4]. The risk of rib fracture increases with age. One study found the incidence of rib fractures among elderly men to be 3.5 per 1000, with 24% of all fractures occurring in osteoporotic men [5]. Another study showed that 56% of patients older than 60 years who died of chest injury suffered no injury worse than a rib fracture [6].

Currently, the gold-standard of treatment is pain management, because pain limits one’s ability to cough and breathe deeply, resulting in sputum retention, atelectasis, and reduced functional residual capacity [7]. These factors can ultimately result in decreased lung compliance, ventilation-perfusion mismatch, hypoxemia, and respiratory distress. Fixation of fractured ribs is controversial, because surgical intervention entails prolonged mechanical ventilation and hospitalization [8]. Other than fixation, however, there is no available treatment aimed at facilitating rib fracture healing. Therefore, multiple rib fractures are serious and constitute a significant unmet clinical need.

Few new ideas have emerged in the biomedical field about how to accelerate bone repair in fractured ribs. Graeber et al. proposed harvesting autografts from a contralateral rib to treat flail chest; they reported improved healing in injured ribs but no healing in donor ribs 30 days after surgery [9]. Santana-Rodriguez and colleagues suggested using ultrasound pulses to facilitate healing in rib fractures, but these researchers reported a nonsignificant increase in bone volume when animals were treated using optimal parameters [10]. None of the osteogenic genes examined in that study was found to be overexpressed, and accordingly none of the treated fractures was reported as healed. Ishihara et al. examined direct gene therapy via an adenoviral vector and indirect gene therapy in which transduced cells were employed to introduce bone morphogenetic protein (BMP) to the injury site. The results﻿, achieved﻿ using an equine model of drilled holes in the rib, were encouraging [11]. However, drilled defect models do not realistically represent the human condition of rib fracture. Adenovirus-mediated gene therapy does carry a promise: researchers found that the Coxsackievirus and adenovirus receptor are specifically expressed in immature osteoblasts residing in fractured ribs and not in healthy intact ribs [12].

Systemically administered mesenchymal stem cells (MSCs) preferentially migrate to sites of injury within the body in various experimental disease models, including myocardial infraction, brain injury, and lung fibrosis models [13]. It has been shown that MSCs home to bones when transplanted into children with severe osteogenesis imperfecta, even in those without recent fractures; this results in denser bone formation and reduced fracture frequency [14]. MSCs can be introduced back into the donor as an autologous graft or used as allogeneic cells to treat other recipients. Since allogeneic MSCs can be preserved as an “off-the-shelf” product and do not require a separate cell isolation phase for each patient, their use is considered advantageous for the clinical setting. Indeed, allogeneic MSCs are being evaluated as a systemic treatment option in a large number of clinical trials. To date, systematic administration of MSCs—autologous or allogeneic—has not yet been investigated for the treatment of rib fractures.

Teriparatide, the 1–34 portion of parathyroid hormone (PTH), has been approved by the US FDA for use as an anabolic agent in the treatment of adults with severe osteoporosis, who are at high risk for fractures. In humans, the anabolic effects of teriparatide manifest as increases in skeletal mass, markers of bone formation and resorption, and bone strength. Preclinical studies also support the potential for PTH as a treatment for bone fractures, demonstrating PTH-improved quality of the fracture callus, increased bone mineral content and density, and accelerated endochondral ossification [15]. Randomized controlled trials aimed at assessing the effect of PTH on fracture healing are ongoing [16]. The PTH anabolic effect stems from its ability to induce the osteoblast transcription factors Osterix (Osx) and Runt-related transcription factor 2 (Runx2) in MSCs [17]. Furthermore, MSCs in treated mice have been shown to progress through accelerated osteoblast maturation and on to accelerated fracture healing concomitant with increased *Osx* expression in fracture calluses. Other studies have demonstrated that PTH stimulates MSC recruitment to bone by inducing C-X-C motif chemokine type 12/stromal cell-derived factor 1 (CXCL12/SDF1) expression in osteoblasts [18, 19]. However, researchers in these preclinical studies used extremely high doses of PTH (approximately 140 times the dose allowed in humans).

We previously studied a combined approach in which we used intravenous (IV) injections of human MSCs (hMSCs) and teriparatide therapy to regenerate vertebral compression fractures using osteoporotic rat and healthy pig models [20]. We showed that teriparatide not only enhanced hMSC homing to vertebral defects by inducing key mediators of MSC migration, but also promoted the terminal differentiation of exogenously delivered cells toward the osteogenic lineage. This dual approach synergistically yielded superior bone formation and fracture repair, compared to each treatment alone, in both animal models. Importantly, the systemically administered hMSCs were undetectable in major organs such as the brain, bone marrow, liver, lung, and spleen 12 weeks after injection. These results of treating vertebral compression fractures using the cell-and-PTH approach are encouraging, although one should bear in mind the unique clinical challenge of multiple rib fractures. The main hurdle preventing a more rapid and effective rib fracture repair is the continual movements and shear forces applied to ribs that inhibit callus formation [21]. In fact, the tensile forces affecting rib fractures are of such magnitude that researchers found in animal studies that the diminished callus that forms is enriched with myofibroblast-like cells [22]. In the present study we hypothesized that systemic administration of hMSCs combined with PTH treatment would enhance cell migration to fractured ribs and induce osteogenic differentiation, which will ultimately lead to effective fracture repair compared to the use of each treatment alone.

## Methods

### Human MSC isolation, expansion, and labeling

Cell preparation was performed in a manner previously described [20]. Briefly, human bone marrow (Lonza, Walkersville, MD, USA) was washed with phosphate-buffered saline (PBS), layered on lymphocyte separation medium, and centrifuged at 900 *g* for 30 min at 30 °C without a break. Mononuclear cells were collected, washed with PBS, and plated at a density of 2 × 10^5^ cells/cm^2^ in media​ supplemented with 10% fetal bovine serum (FBS, Invitrogen, Carlsbad, CA, USA), in 5% CO^2^/95% air at 37 °C. The cells were cultured until they reached the fifth passage and then were prestained with a lypophilic fluorescent dye (Vybrant-CM-DiI, Invitrogen) prior to in vivo injection.

### Multiple rib defect surgery

**Fig. 1:**
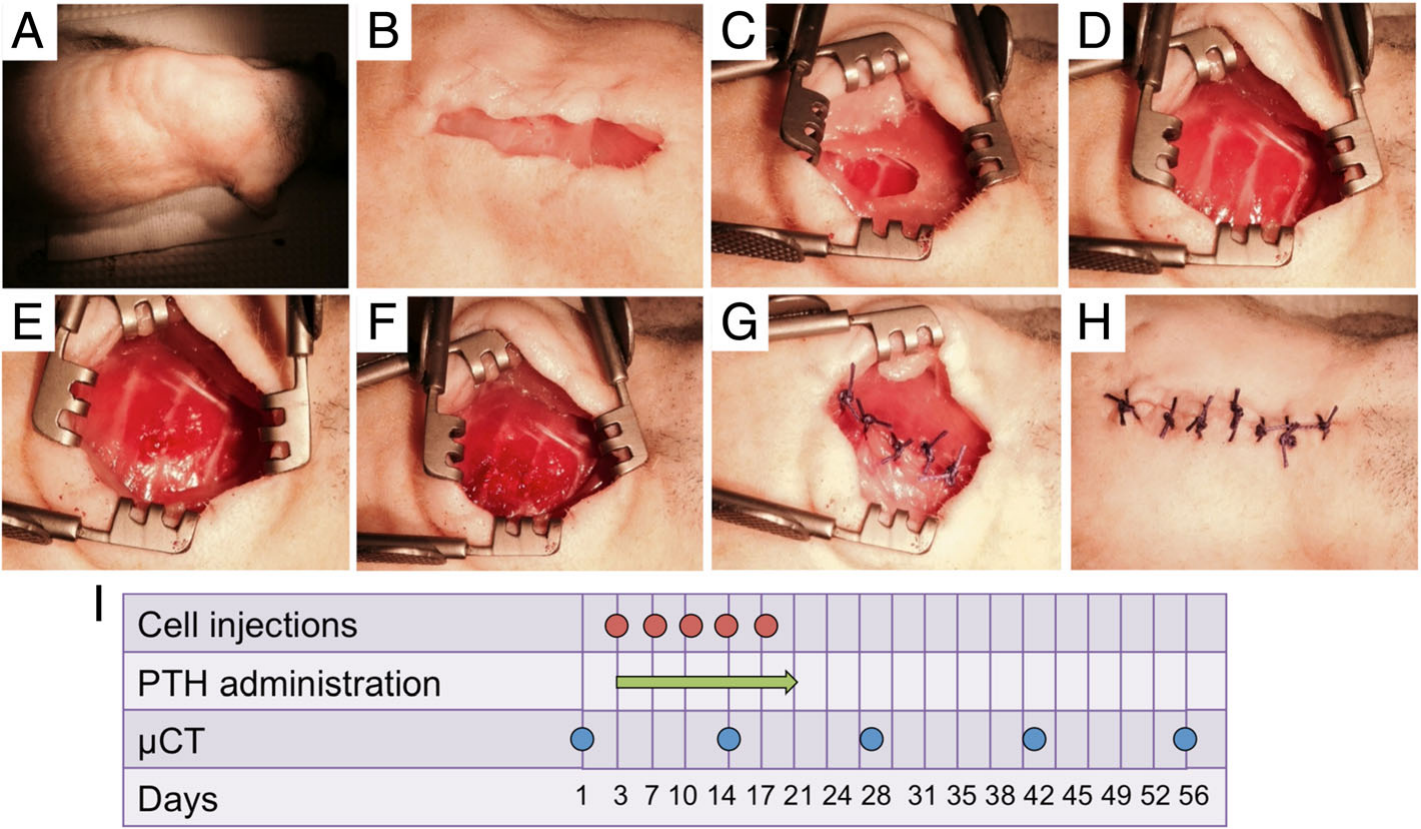
Rib defect surgery and timeline. After the rat had been anesthetized, intubation and mechanical ventilation were established to maintain positive pleural pressure. The animal was then placed on its side and shaved (**a**). A 2-cm-long longitudinal cut was made along the mid-axillary line (**b**), and the latissimus dorsi muscle was removed by blunt dissection (**c**) until the rib cage was fully exposed (**d**). A 5-mm-long segment was removed from both the fifth (**e**) and sixth (**f**) ribs. The ribs were stitched separately, followed by suturing of the muscular layer (**g**) and skin (**h**). The animals were divided into four groups: (1) a negative control group; (2) the hMSC-only group, in which rats received five injections of 2 × 10^6^ hMSCs every 3–4 days beginning on the 3^rd^ day after surgery; (3) the PTH-only group, in which rats received daily injections of 4 μg/kg PTH for 17 days, beginning at the third day after surgery; and (4) the hMSC + PTH group, in which rats received both treatments. The animals were scanned by μCT biweekly until they were sacrificed 8 weeks after surgery (**i**). *PTH* parathyroid hormone, *μCT* micro-computed tomography

All procedures described in this study were approved by the Cedars-Sinai Medical Center’s Institutional Animal Care and Use Committee (Request No. 5684). The animals were treated in strict adherence to NIH guidelines. Thirty-six 12-week-old female athymic rats (Crl:NIH-Foxn1^rnu^) were purchased from Charles River Laboratories (Wilmington, MA, USA). The animals were anesthetized by intraperitoneal injections of ketamine/dexmedetomidine, after which they received intubation and mechanical ventilation (Model 683 small animal respirator, Harvard Apparatus, Holliston, MA, USA), to establish positive pressure respiration. Each animal was placed in the lateral position, and the chest area was shaved and disinfected using iodine and chlorhexidine (Fig. 1a). A 2-cm-long incision was made along the lateral aspect of the thorax through the skin, subcutaneous tissue, and the latissimus dorsi muscle, exposing the rib cage (Fig. 1b–d). A 5-mm-long segment was excised from the bony portion of the fifth rib, avoiding damaging the rib’s cartilaginous portion (Fig. 1e). Next the periosteum was removed and the intercostal muscles were sutured using a 5-0 Vicryl suture. A defect was created in the sixth rib in a similar manner (Fig. 1f). The muscular layer and skin were then sutured separately (Fig. 1g, h), and the animal was removed from mechanical ventilation and anesthesia. After the procedure, each rat was randomly assigned to one of the following experimental groups (eight to ten rats per group): (1)the negative control group, in which animals were given only vehicle (PBS); (2) the hMSC-only group, in which each animal received separate IV injections of 2 × 10^6^ hMSCs on days 3, 7, 10, 14, and 17 after surgery; (3) the PTH-only group, in which animals received daily subcutaneous injections of PTH (4 μg/kg body weight/day; Teriparatide, Forteo, Eli Lilly, Indianapolis, IN, USA) for 17 days beginning on day 3; and (4) the hMSC + PTH group, in which animals received the combined therapy of hMSCs and PTH (Fig. 1i).

### Longitudinal micro-computed tomography (μCT) imaging of bone formation

The ribs were evaluated using a cone-beam in vivo μCT imaging system (vivaCT 40; Scanco Medical, Brüttisellen, Switzerland). To analyze healing, the animals were imaged on day 1 and again 2, 4, 6, and 8 weeks after generation of the bone defect. Microtomographic slices were acquired using an X-ray tube with a 55-kVp potential and an intensity of 145 μA, and reconstructed at a voxel size of 35 μm. Histomorphometric three-dimensional (3D) evaluation was performed following definition of a volume of interest (VOI) that included the bony defect margins as well as the area from which the bone was resected; a constrained 3D Gaussian filter (σ = 0.8, support = 1) was used to partly suppress the VOI noise. The bone tissue was segmented from marrow and soft tissue by using a global thresholding procedure in which 200 mgHa/cm^3^ was determined to be the threshold as it matched the histological rib morphology. For each rib, the defect margins were located on day 1 scans and aligned to a standard position [23]; a cylindrical VOI (5.25 mm in diameter, 7 mm in height) was defined for 3D histomorphometric evaluation. The anatomical match obtained by the registration procedure allowed us to apply the predefined VOI of day 1 to all remaining time points. Bone volume density was used to assess new bone formation. In addition, the percentage of completely gapped ribs was calculated for each group.

### Biomechanical analysis of ribs

Micro-finite element (μFE) analysis was used to compare the biomechanical characteristics of healed ribs to intact ribs when subjected to compression, torsion along the rib axis, and bending of the healed segment in two perpendicular planes. For each healed rib (revealed by μCT scanning), a consecutive rib from the same animal was chosen as a control. Each rib was aligned to the standard position as described above, and regions of low, medium, and high mineralization were defined by threshold as 200 mgHa/cm^3^, 500 mgHa/cm^3^, and 800 mgHa/cm^3^, respectively. Each voxel was converted into an equally shaped brick element for the μFE model. The low, medium, and high areas of mineralization were assigned a Young modulus of 50 MPa, 5 GPa, and 15 GPa, respectively. In all cases, these boundary conditions were applied by prescribing displacements of the nodes at the top and bottom surfaces in such a way that a longitudinal compressive strain of 1% or an angular rotation at the long ends of 0.01 radians results. Stiffness was then calculated as the ratio of the reaction force over the applied displacement (compression, N/mm) [24], or the ratio of the reaction moment over the applied angular rotation (torsional, x bending, and y bending, Nmm/rad) [25]. Analysis was done by Scanco Medical, Zurich, Switzerland.

### Histological analysis and immunofluorescence imaging

Histological analysis was performed on rat ribs that had been retrieved 8 weeks postoperatively (the endpoint of the experiment). The ribs were sectioned along the longitudinal axis, preserved using paraformaldehyde, and embedded in paraffin. Five-micrometer slices were sectioned, deparaffinized, and stained using hematoxylin and eosin (H&E) for a morphological analysis, as previously described [20]. For immunofluorescence staining, the tissues were deparaffinized, and the antigens were retrieved by incubation in preheated Target Retrieval Solution (Dako, Carpinteria, CA, USA) for 45 min at 37 °C. Nonspecific antigens were blocked by applying serum-free blocking solution (Dako). Tissue slides were stained with primary antibodies against human bone sialoprotein (BSP) and osteocalcin (Oc) to examine osteogenic differentiation and with Stromal cell-Derived Factor 1 (SDF1), C-X-C motif receptor type 4 (CXCR4), epidermal-like growth factor receptor (EGFR), and amphiregulin (Amp) antibodies to determine the mechanism of MSC migration. These primary antibodies were applied to the slides and incubated at 4 °C overnight, after which they were washed off using PBS. Then the slides were incubated with secondary antibodies (Additional file 1: Table S1) for 1 hour at room temperature; these antibodies were also washed off with PBS. The slides were stained with 1 μg/ml 4′,6-diamidino-2-phenylindole dihydrochloride (DAPI) for 5 min in the dark, after which they were finally washed three times with PBS. VectaMount mounting medium (Vector Laboratories, Burlingame, CA, USA) was applied to the tissue. Tissue samples on the slides were imaged using a four-channel confocal Laser Scanning Microscope 780 (Zeiss, Pleasanton, CA, USA) with × 20 magnification, z-stacking, and 5 × 5 tile scanning. For zoom-in images, a single z-stacked image was generated. All samples were scanned using the same gain and exposure settings. In addition, DiI-positive cells in five fields of × 40 magnification were automatically counted using ImageJ software.

### Statistical analysis

GraphPad Prism 5.0b software (GraphPad Prism, San Diego, CA, USA) was used to analyze the data. The results are presented as means ± SE (^*^ *p* ≤ 0.05; ^**^ *p* ≤ 0.01; ^***^ *p* ≤ 0.001; ^****^ *p* ≤ 0.0001; ns = not significant). The longitudinal data analysis was conducted using a one-way ANOVA or a two-way ANOVA with repeated measures and the Bonferroni post-test. To assess significance, *p* < 0.05 was considered statistically significant.

## Results

### PTH enhances hMSC migration to the rib fracture

**Fig. 2:**
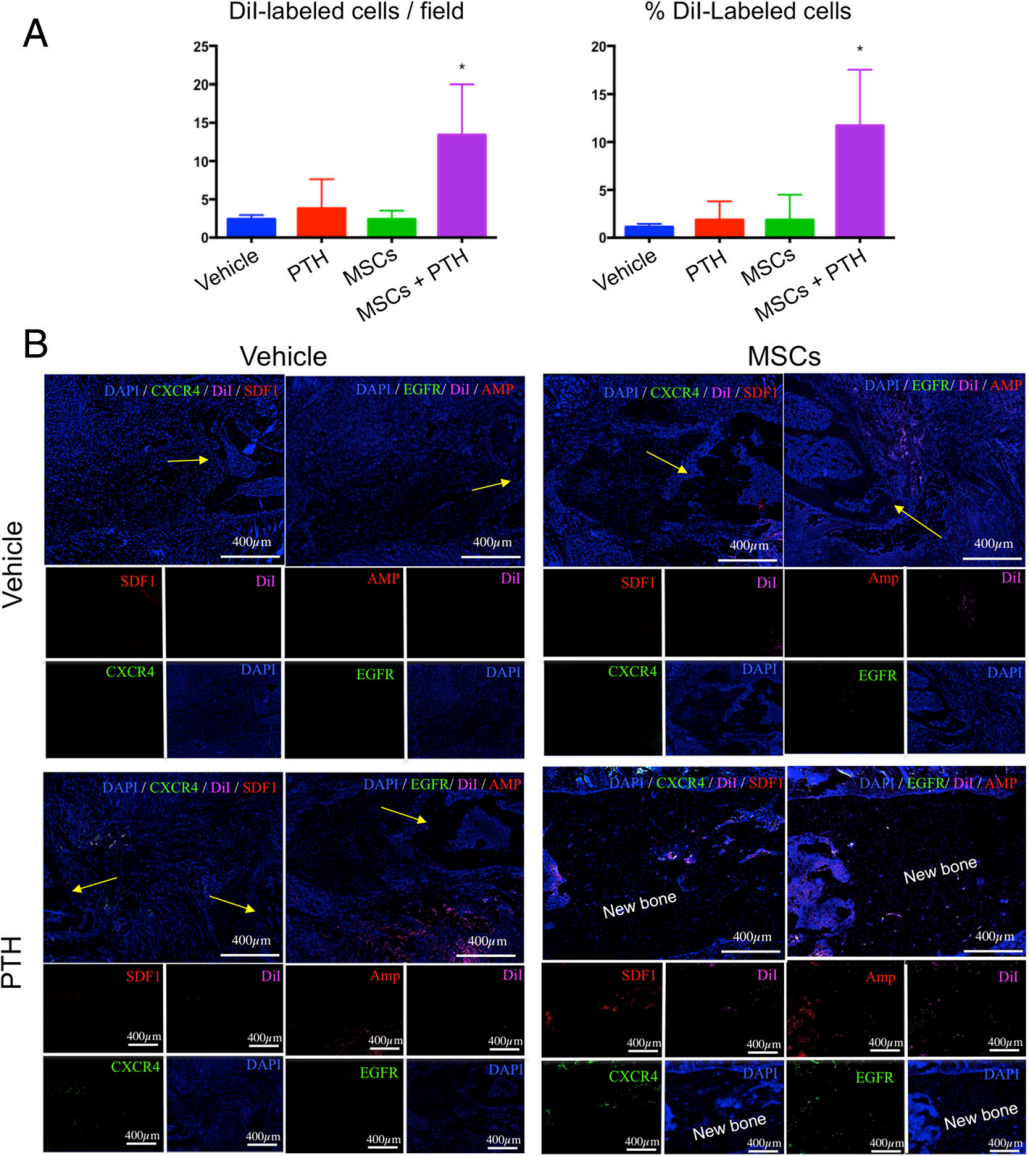
PTH enhances cell migration to the fractured rib. Rats were sacrificed 8 weeks after surgery. Chest wall samples were harvested and processed for histological labeling of hMSCs to allow quantification of cell homing to rib fractures. Tissue slides were scanned using confocal microscopy imaging, and the DiI-positive cells were counted with the aid of ImageJ software. The percentage of DiI-positive cells among DAPI-positive cells was calculated as well (**a**) (^*^ *p* ≤ 0.5, two-way ANOVA, *n* = 5). A qualitative analysis was performed on slides stained for stromal cell-derived factor 1 (*SDF1*), C-X-C chemokine receptor type 4 (*CXCR4*), epidermal growth factor receptor (*EGFR*), amphiregulin (*Amp*), and the *D﻿iI*-stained transplanted ﻿MSCs (**b**). *Yellow arrows* indicate bone defect margins. *MSCs* mesenchymal stem cells, *PTH* parathyroid hormone

We were able to quantify the homing of exogenously delivered hMSCs to the fracture site by histological analysis, thanks to pre-labeling of the cells with the lipophilic fluorescent dye DiI. The animals were sacrificed 8 weeks after surgery and histological processing was performed. Significantly more DiI-stained cells were counted in ribs﻿ of﻿ animals given both hMSCs and PTH than in animals given hMSCs only (Fig. 2a): 15 DiI-positive cells per × 40 field compared to 5 DiI-positive cells, respectively. When we looked exclusively at DAPI-positive cells in all groups, we calculated the percentages of DiI-positive cells to be on average 12% in the hMSC + PTH group and only 2.5% in the hMSC group—no more than the percentages of DiI-positive cells among DAPI-positive cells found in the other groups, which had not been given any cells at all (i.e. background staining). Next we examined activation of the stromal cell-derived factor 1 (SDF1)/C-X-C chemokine receptor type 4 (CXCR4) axis, a critical pathway of MSC recruitment in the fracture-healing process (Fig. 2b) [26, 27]. We found ample immunostaining of both SDF1 and CXCR4 in the hMSC + PTH group and, only in that group, in the area of new bone formation. An alternative pathway of MSC mobilization that is induced by PTH treatment is the pathway of amphiregulin, an epidermal growth factor (EGF)-like ligand that is secreted from injured tissue and signals through the epidermal growth factor receptor (EGFR) in homing cells [28]. We found prominent staining of amphiregulin in both groups of animals that received PTH therapy (Fig. 2b). In the hMSC + PTH group, we found EGFR staining co-localized with DiI staining, signifying systemically administered hMSCs (Fig. 2b).

### PTH therapy induced osteogenic differentiation in hMSCs in vivo

**Fig. 3:**
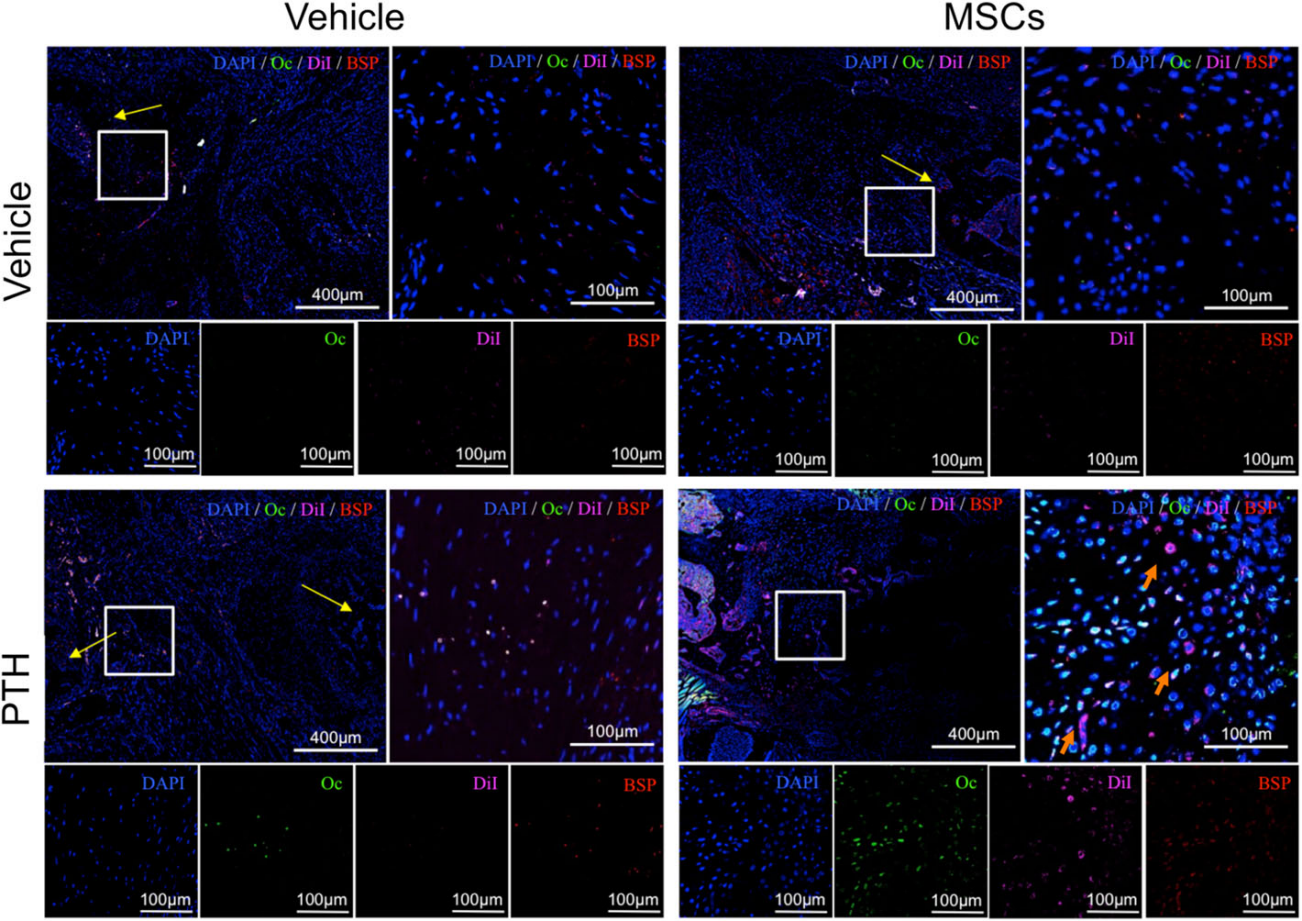
PTH induces hMSC differentiation into osteoprogenitor cells. Immunohistochemistry was performed on tissue samples harvested 8 weeks after surgery. The sections were stained with antibodies against the well-established osteogenic markers osteocalcin (*Oc*) and bone sialoprotein (*BSP*), in addition to *DAPI* staining. *Yellow arrows* indicate bone defect margins; *orange arrows* indicate co-staining of *DiI* and bone markers. *MSCs* mesenchymal stem cells, *PTH* parathyroid hormone

We stained tissue sections with primary antibodies against two well-established markers of osteogenesis, bone sialoprotein (BSP) and human osteocalcin (Oc) [29], to detect osteogenic differentiation in injected hMSCs (Fig. 3). Our results showed that, indeed, PTH induced osteogenic differentiation in systemically injected hMSCs (Fig. 3). In the negative control group and in the hMSC-only group there was scarce staining of osteogenic markers, whereas in the PTH-only group some Oc staining was present. In the hMSC + PTH group, we observed ample Oc and BSP staining. Both markers were found in DiI-positive cells, and Oc was detected in many non–DiI-positive cells as well.

### Systemic administration of both hMSCs and PTH induces superior healing of rib fractures

**Fig. 4:**
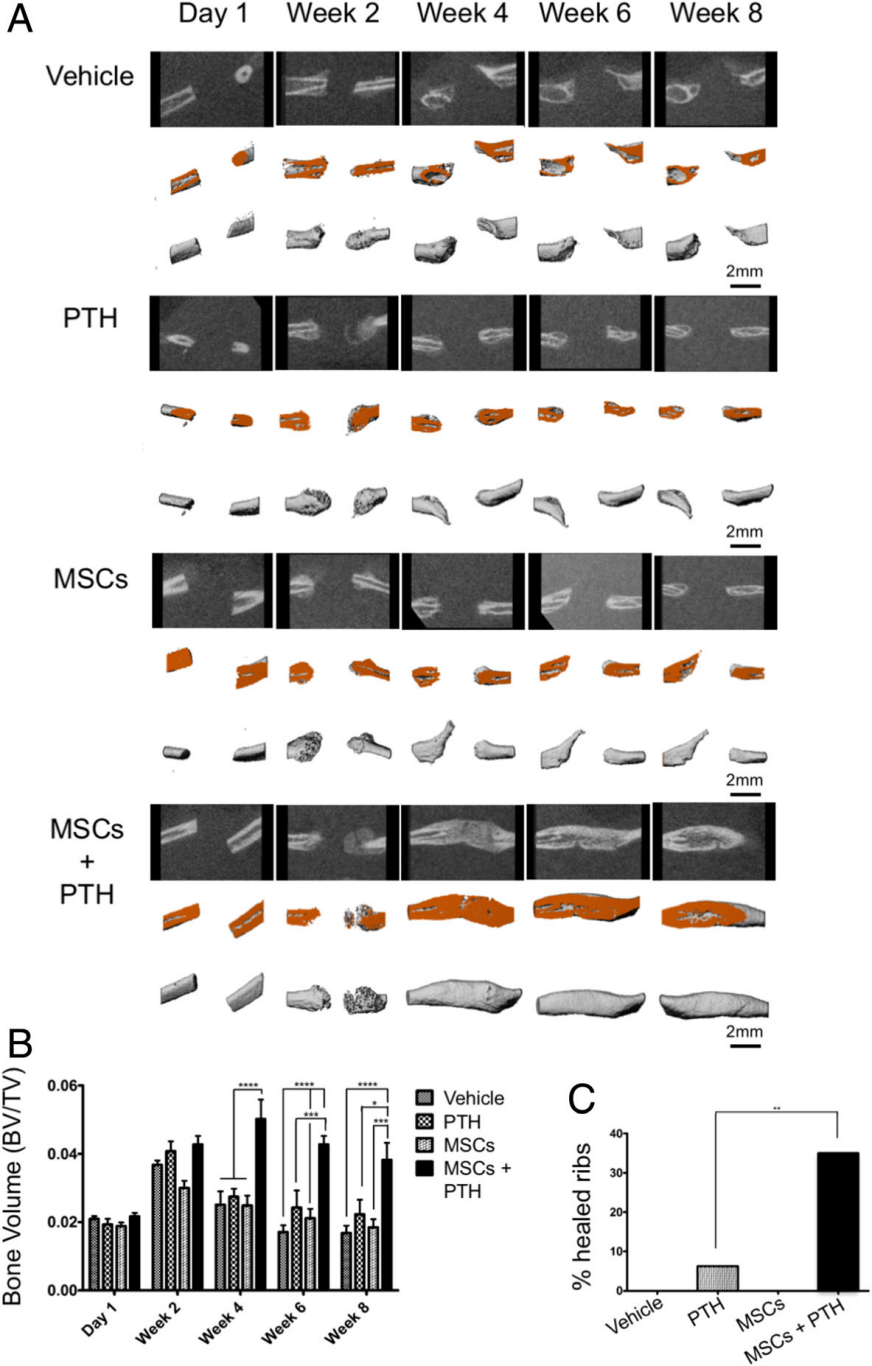
hMSC + PTH combined therapy induces superior bone formation that leads to bridging of rib fractures: micro-computed tomography analysis. In vivo μCT scans were obtained biweekly. Each rib sample was aligned to a standard position, and a cylindrical volume of interest (VOI) was defined following separation of the bone from soft tissue (**a**). For each experimental group, the *top-row images* present 2D reconstructions, sectioned in the rib midline. The *middle-row images* present 3D reconstructions, cut in the corresponding plane (cut-plane marked with *orange). Lower-row images* present the complete 3D reconstruction of the analyzed VOI. The bone volume was quantified (**b**) (Two-way ANOVA, *n* = 8–10). Percentages of healed ribs by the 8^th^ week were measured manually and calculated for each group (**c**). χ^2^(3,72) = 16.44, *n* = 8–10). *MSCs* mesenchymal stem cells, *PTH* parathyroid hormone

**Fig. 5:**
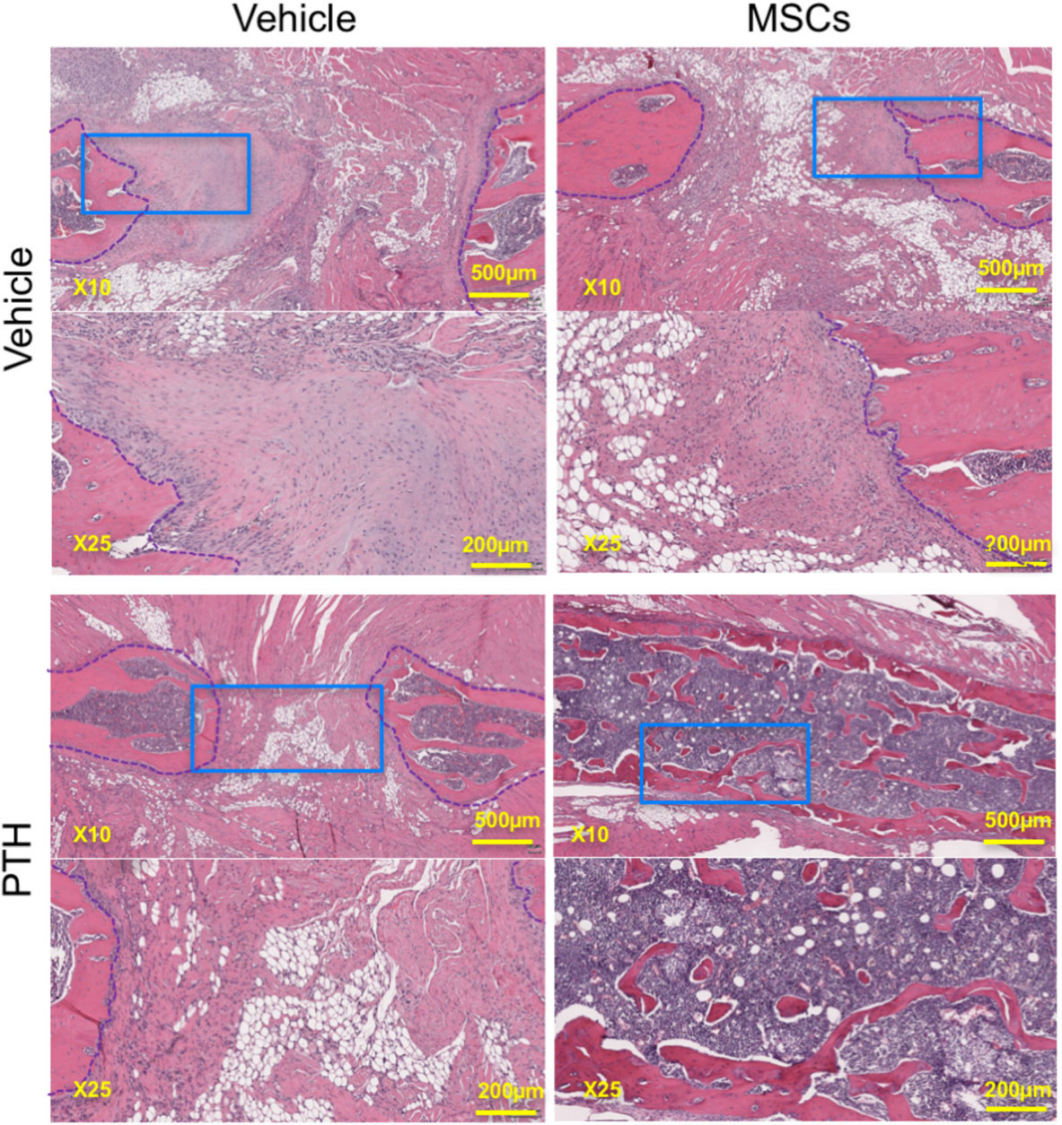
hMSC + PTH combined therapy induces superior bone formation that leads to bridging of rib fractures: histological analysis. Samples were harvested 8 weeks after creation of multiple rib fractures, and prepared by standard formaldehyde fixation. Analysis was performed using H&E staining (margins of the segmental rib defect are marked with *blue dashed lines. Light blue rectangles* in the × 10 images mark the corresponding area that is enlarged in the × 25 images). *MSCs* mesenchymal stem cells, *PTH* parathyroid hormone

To assess bone formation induced by the combined cell-and-PTH approach, we performed μCT biweekly. Two weeks after the multiple rib fractures were induced, qualitative μCT analysis demonstrated prominent bone formation stemming from the bone stumps in the animals given PTH-alone and MSC + PTH treatment (Fig. 4a). Two weeks later, the newly formed bone was resorbed in the PTH-only group, while bone calluses bridging the defects were observed in several animals given MSC + PTH. By the 6^th^ and the 8^th^ week after surgery, these bone calluses remodeled so that intramedullary soft tissue could be discerned from mature cortexes. By the latter time point, limited bone regeneration beyond the defect margins was clearly demonstrated in all other experimental groups. Quantitative analysis demonstrated the formation of significantly more bone volume in the hMSC + PTH group beginning in the 4^th^ week (Fig. 4b); this continued to be the case until the end of the experiment, 8 weeks after surgery. In all experimental groups, other than the hMSC-only group, we observed insignificant increases in bone volume in the 2^nd^ week. By the 4^th^ week, bone volumes in the negative control group (which received vehicle only) and PTH-only group returned to the levels of bone volume measured a day after the defects had been created. At the same time point there was twice as much bone volume in the hMSC + PTH group. The percentages of healed ribs in all groups were calculated based on careful examinations of 2D and 3D μCT reconstructions (Fig. 4c); in the negative control and hMSC-only groups no ribs healed. In the PTH-only group we found bone bridging in one rib (1/16 ribs, 6.25%), while in the hMSC + PTH group we found bone bridging in 7 of 20 (35%). Interestingly, fractured ribs that displayed no bone bridging or near bone bridging by the 4^th^ week did not achieve bridging at any later time point (data not shown). In the hMSC + PTH group, histological analysis using H&E staining confirmed bone bridging and revealed a trabecular structure of woven bone filled with vital bone marrow (Fig. 5). In the other experimental groups, particularly in the hMSC-only and negative control groups, we detected fibrotic tissue between the defect margins.

### Healed ribs are stiffer than intact ribs

**Fig. 6:**
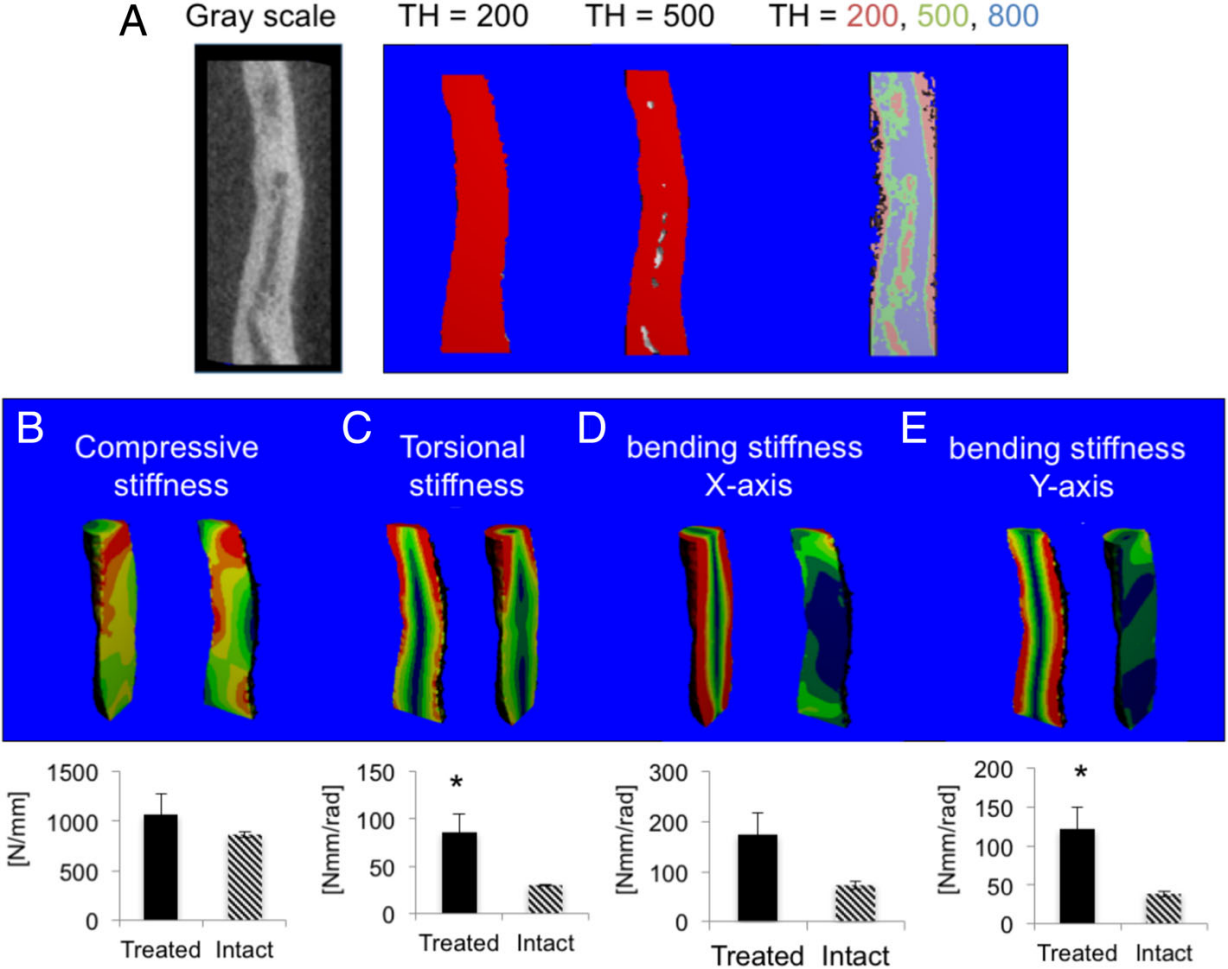
Healed ribs are stiffer than intact ribs, indicating a remodeling bone callus. μCT scans were further evaluated using virtual biomechanical testing using μFE analysis. Low mineralization was defined as greater than 200 mgHa/cm^3^; medium mineralization as greater than 500 mgHa/cm^3^; and high mineralization as greater than 800 mgHa/cm^3^ (**a**). Low mineralization is marked *red*; medium mineralization, *green*; and high mineralization, *blue*. Each voxel was converted to a μFE brick, and compression of the sample was simulated (**b**). To examine torsion and bending, the x-axis was defined as bending in the plane of the chest wall, while the y-axis was defined as bending outwardly (or inwardly toward the lungs). An angular twist was stimulated to simulate torsion powers (**c**), and bending was simulated along the x (**d**) and y (**e**) axes. *TH* threshold

To evaluate the quality of rib healing beyond the mere finding of gap bridging, we performed virtual biomechanical testing using a micro-finite element method analysis (μFE). For this purpose, we converted each μCT voxel into a brick element and used multiple thresholds—200, 500, and 800 mgHa/cm^3^—to realistically mimic levels of strain on the bone (Fig. 6a). The healed rib stiffness with respect to compression was similar to that of intact ribs, about 1000 N/mm (Fig. 6b). Stiffness in response to torsion was significantly higher in the healed rib group: 90 Nmm/rad compared to 30 Nmm/rad. When we measured stiffness in response to bending on the x-axis, upward towards the consecutive rib in the plane of the chest wall, healed ribs yielded a measurement of 200 Nmm/rad compared to 90 mgHa/cm^3^ in intact ribs. Bending along the y-axis outwardly (or inwardly towards the lungs) yielded a significant change in stiffness: 150 Nmm/rad in healed ribs compared to 30 mg Ha/cm^3^ in intact ribs.

## Discussion

Systemic administration of MSCs has been proposed to facilitate regeneration of various injured tissues including the myocardium [30], spinal cord [31], and the brain [32], in which the involvement of the SDF1/CXCR4 axis has been demonstrated [33]. These reports are in accordance with our findings (Fig. 2b), and also with a previously published study on bone regeneration [34]. Intravenous administration is the most common approach to the circulatory system, and the main hurdle is that injected cells must pass through the lungs prior to their distribution throughout the body [35]. Several methods have been proposed to enhance MSC migration to targeted organs, including magnetic guidance of cells pre-labeled with super-paramagnetic iron oxide nanoparticles [36, 37], coating cells with vascular cell adhesion molecule antibodies [38], and also the use of a synthetic peptide-mimetic ligand (which has a high affinity for activated α4β1 integrin on the surface of MSCs) conjugated to a bisphosphonate in order to target MSCs to the osteoblast surface [39].

The optimal targeting strategy will use a device or a drug that enhances both MSC targeting and differentiation to achieve fracture healing. PTH, the only anabolic treatment for osteoporosis in postmenopausal women that is approved by the FDA [40], has been found in both animal and clinical research to enhance fracture healing [41]. PTH treatment increases SDF-1 mRNA levels near epiphyseal plates while significantly decreasing serum SDF-1 levels, creating a gradient that possibly enhances cell engraftment [18]. As mentioned above, we have demonstrated that PTH promotes exogenous MSC homing to vertebral fractures in models of osteoporotic rats and healthy pigs by activation of the SDF1/CXCR4 and amphiregulin/EGFR axes [20]. PTH therapy induced expression of Oc and BSP, which led to complete regeneration of injured vertebrae that was more significant than any treatment alone.

Given those findings, we decided to examine the ability of the combined cell-and-PTH approach to regenerate multiple rib fractures in a rat model. In the present study, we addressed the clinical need of multiple rib fracture regeneration. Our results demonstrate that migration of systemically administered hMSCs to the fractured rib is enhanced by intermittent PTH treatment via activation of two parallel pathways: SDF1/CXCR4 and amphiregulin/EFGR (Fig. 2). Interestingly, we observed a population of CXCR4-positive cells in the ribs of animals that were given PTH treatment alone. Endogenous CXCR4-expressing cells were not observed in the vertebral model in which we used osteoporotic ovariectomized rats [20], which can be explained by the report that ovariectomized rats have diminished MSC population in bone marrow available to respond to PTH signaling [42]. We showed that PTH therapy induces stem cell differentiation (Fig. 3), which leads to greater bone formation and a higher rate of bone bridging across the defect when the combined hMSC-PTH therapy is given (Figs. 4 and 5).

While some exogenously administrated DiI-positive cells were found to express osteocalcin and bone sialoprotein under PTH induction, representing osteogenic differentiation, many non-DiI host cells were found to express these osteogenic markers in the MSC + PTH group. The enhanced recruitment and differentiation of resident cells that was not observed when only cells were administrated and only scarcely observed when PTH was given alone, can be explained by MSC paracrine effects, which play a central role in response to injury [43]; specifically, by exosomal signaling that has been shown to play a key role in bone marrow MSC differentiation [44] and in fracture healing [45].

Lastly, we demonstrated that using this combined therapy, healed ribs are stiffer than intact ribs when subjected to torsion and bending along the plane perpendicular to the chest wall (Fig. 6). This last point is of importance to the patient, because stiffer ribs are less likely to move and cause pain while breathing or coughing [46]. The stiffness is typical to bone repaired by stem cell therapy, and the ribs are expected to remodel over time evolving to be less dense, allowing some elasticity [24, 47]. Interestingly, simple fracture repair involves formation of a bone callus which is softer than native bone and does not remodel to a less stiff bone [48, 49], though Manjubala et al. reported that the bone callus reaches its maximum stiffness 6 weeks after fracture and becomes softer after 9 weeks [50]. Taken together, the predicted stiffness of the rib bone callous may be attributed to the artificial biological enhancement of fracture repair by MSC and PTH, and an interesting future research would be the examination of repaired rib biomechanics over time.

We initially performed a pilot study in which we used a single-point fracture that had been created by tweaking the rat rib with a bone cutter. These fractures healed by the 4^th^ week in all experimental groups, regardless of the given treatment (data not shown). We concluded that the single-point fracture does not realistically mimic the clinical event, in which rib fractures heal only after a prolonged time, and thus we switched to a segmental defect model. We excised 5-mm-long segments from the fifth and sixth ribs, and noted that a day later, the gap was about 1–2 mm long, which may be attributed to collapse of the chest wall (Fig. 4a). We also noted that although the defect margins were not aligned at that time point, 8 weeks later they were in all animals, even when bridging did not occur. Future studies of these interesting phenomena may explain how bone bridging is achieved in a continual moving environment. In two animals, bone formed between the fifth and sixth ribs’ dorsal margins and between the fifth and sixth ribs’ ventral margins, from which we can infer that there is some paracrine signaling (data not shown). One limitation of our model is that we had to resect the periosteum, as it is known to induce spontaneous healing in a 5-mm segmental rib defect [51]; which does not truly mimic the clinical condition. Another limitation is the fact that our treatment protocol yielded a union rate of 35% (Fig. 4c), which although higher than any union rate obtained using other strategies reported above, is not optimal and necessitates refinement of the protocol. Optimization of PTH dosing and cell administration is central to this purpose. A PTH dosage of 4–40 μg/kg per day is generally used in rodent studies [52, 53]; the FDA-approved PTH dose for human patients is 20 mg/day, which in rats is approximately equivalent to 0.3–0.5 μg/kg/d. Higher doses do not necessarily lead to improved results, and looking at the rat vertebral fracture model we found that the 0.4 μg/kg dose yields a faster increase in bone density, suggesting that human dosing is preferable [20]. The ideal systemic cell administration protocol would include a single systemic injection for the sake of patient comfort. Kean et al. have demonstrated the efficiency of intra-arterial MSC injection, which represents a promising future direction [35]. Another approach to enhance cell homing would be intra-cardiac administration, that may lead to tenfold more cells arriving to the defect site [54].

To evaluate the true impact of our proposed approach on patient recuperation, future studies of the cell-and-PTH treatment’s effect on pain and respiration should be performed. The rodent model we describe allows the use of relatively easy and accurate methods to study these key parameters. For example, respiration dynamics can be quantified by using whole body plethysmography, and any pain experienced by the rats can be evaluated using well-established behavioral tests such as the rotating rod and open field tests.

## Conclusions

Intermittent PTH treatment has promoted the migration of systemically administrated hMSCs and induced osteogenic differentiation, leading to rib fracture repair and gap bridging. The success of the cell-and-PTH approach could lead to a novel stem cell therapy for life-endangering ribs fractures, and may prove beneficial for other clinical indications such as craniofacial and carpal fractures.

## Additional files

Additional file 1: Antibodies and Dilutions utilized throughout the study. (DOCX 86 kb)

## Abbreviations

3D: Three dimensional
Amp: Amphiregulin
BSP: Bone sialoprotein
CXCL12: C-X-C motif chemokine type 12, also known as stromal cell-derived factor 1 (SDF1)
CXCR4: C-X-C motif receptor type 4
DAPI: 4′,6-Diamidino-2-phenylindole dihydrochloride
EGFR: Epidermal-like growth factor receptor
FBS: Fetal bovine serum
H&E: Hematoxylin and eosin
hMSC: Human mesenchymal stem cell
IV: Intravenous
Oc: Osteocalcin
Osx: Osterix
PBS: Phosphate-buffered saline
PTH: Parathyroid hormone
Runx2: Runt-related transcription factor 2
SDF-1: Stromal cell-derived factor 1, also known as C-X-C motif chemokine type 12 (CXCL12)
VOI: Volume of interest
μCT: Micro-computed tomography
μFE: Micro-finite element (method)
