# Supplementary material for: Systemic administration of mesenchymal stem cells combined with parathyroid hormone therapy synergistically regenerates multiple rib fractures
Source: Stem Cell Res Ther. 2017 Mar 9;8:51. doi: 10.1186/s13287-017-0502-9 (PMC5345153; doi:10.1186/s13287-017-0502-9)
Supplement: Additional file 1: — Antibodies and Dilutions utilized throughout the study. (DOCX 86 kb) [file 13287_2017_502_MOESM1_ESM.docx]

Systemic administration of mesenchymal stem cells combined with parathyroid hormone therapy synergistically regenerates multiple rib fractures

**Supplemental Materials**

**Antibodies and Dilutions utilized throughout the study**

| **Antigen** | **1’ antibody** | **1’ antibody dilution** | **2’ antibody *** | **2’ antibody dilution** |
| --- | --- | --- | --- | --- |
| Bone Sialoprotein | BSP, mouse anti-human, Millipore, Temecula, CA , Clone-ID1.2, cat#203637-100ug | 1:250 in 3% Donkey serum and 0.3% Triton X PBS | Cy5-conjugated donkey anti-mouse antibody, polyclonal, cat#715-175-150 | 1:1000 in 0.3% PBS-T |
| CXCR4 | Goat anti human/rat CXCR4, Abcam, polyclonal, cat#AB1671 |  | [Alexa Fluor® 647](http://www.abcam.com/index.html?pageconfig=resource&rid=15841) donkey anti-goat antibody, Polyclonal, cat#705-605-003 |  |
| Osteocalcin | Oc, rabbit anti human/rat, Millipore, Polyclonal, cat#AB10911 |  | Alexa Fluor® 488 donkey anti-rabbit polyclonal cat# 711-545-152 |  |
| SDF1 | Rabbit anti human/rat, Abcam, Cambridge, MA, polyclonal, Cat#AB9797 |  |  |  |
| Amphiregulin | Amp, rabbit anti-human/rat, Bioss, Woburn, MA, polyclonal, cat#bs-3847R | 1:200 in 3% Donkey serum and 0.3% Triton X PBS |  | 1:500 in 0.3% PBS-T |
| EGFR | Mouse anti human EGFR, BioLegend, San Diego, CA, Clone-AY13, cat#352902 |  | Cy5-conjugated donkey anti-mouse antibody, Polyclonal, cat# 715-175-150 |  |

**Table 1.** Antibodies used for the immunofluorescence throughout the study. *Purchased from Jackson ImmunoResearch Laboratories, West Grove, PA
